# Continuous Subcutaneous Insulin Infusion in Children: A Pilot Study Validating a Protocol to Avoid Hypoglycemia at Initiation

**DOI:** 10.3389/fendo.2017.00084

**Published:** 2017-04-24

**Authors:** Despoina Manousaki, Johnny Deladoëy, Louis Geoffroy, Patricia Olivier

**Affiliations:** ^1^Endocrinology Service and Diabetes Unit, CHU Sainte-Justine, Montreal, QC, Canada; ^2^Department of Pediatrics, University of Montreal, Montreal, QC, Canada

**Keywords:** continuous subcutaneous insulin infusion, pediatrics, hypoglycemia, continuous glucose monitoring, type 1 diabetes

## Abstract

**Background:**

The occurrence of hypoglycemia and hyperglycemia during the first days after transition to continuous subcutaneous insulin infusion (CSII) in patients with type 1 diabetes has not been systematically studied in children. The aim of this prospective study was to demonstrate that the protocol applied in our diabetes clinic is safe at CSII initiation in children.

**Methods:**

We assessed 22 pediatric patients with type 1 diabetes, using continuous glucose monitoring (CGM) before and after CSII initiation (±3 days).

**Results:**

After CSII initiation, there was no difference in the rates of hypoglycemic events expressed as relative rates (RRs) per person-reading (RR = 0.85, *p* = 0.52, 95% CI 0.52–1.39), as well as in the number of prolonged hypoglycemic events (>1 h) per day (RR = 1.12, *p* = 0.56, 95% CI 0.75–1.68). We observed only a trend toward prolonged episodes of hyperglycemia after pump initiation (RR = 1.52, *p* = 0.06, 95% CI 0.97–2.35).

**Conclusion:**

Our study is the first to assess, through CGM and in a prospective way, the impact of a CSII initiation protocol on glycemic values. Our protocol provides a safe model to avoid hypoglycemia at CSII initiation in children.

**Clinical Trial Registration:**

www.ClinicalTrials.gov, identifier NCT01840358.

## Introduction

Continuous subcutaneous insulin infusion (CSII) is a highly accurate and flexible tool for administration of insulin. However, good glycemic control is often difficult to achieve even with CSII. Although previous pediatric and adult studies provided evidence of an increased risk of hypoglycemia with CSII (1–5), recent studies in children have documented a decrease in this risk (6–9) with an increase in the risk of episodes of ketoacidosis (7).

**Figure 1:**
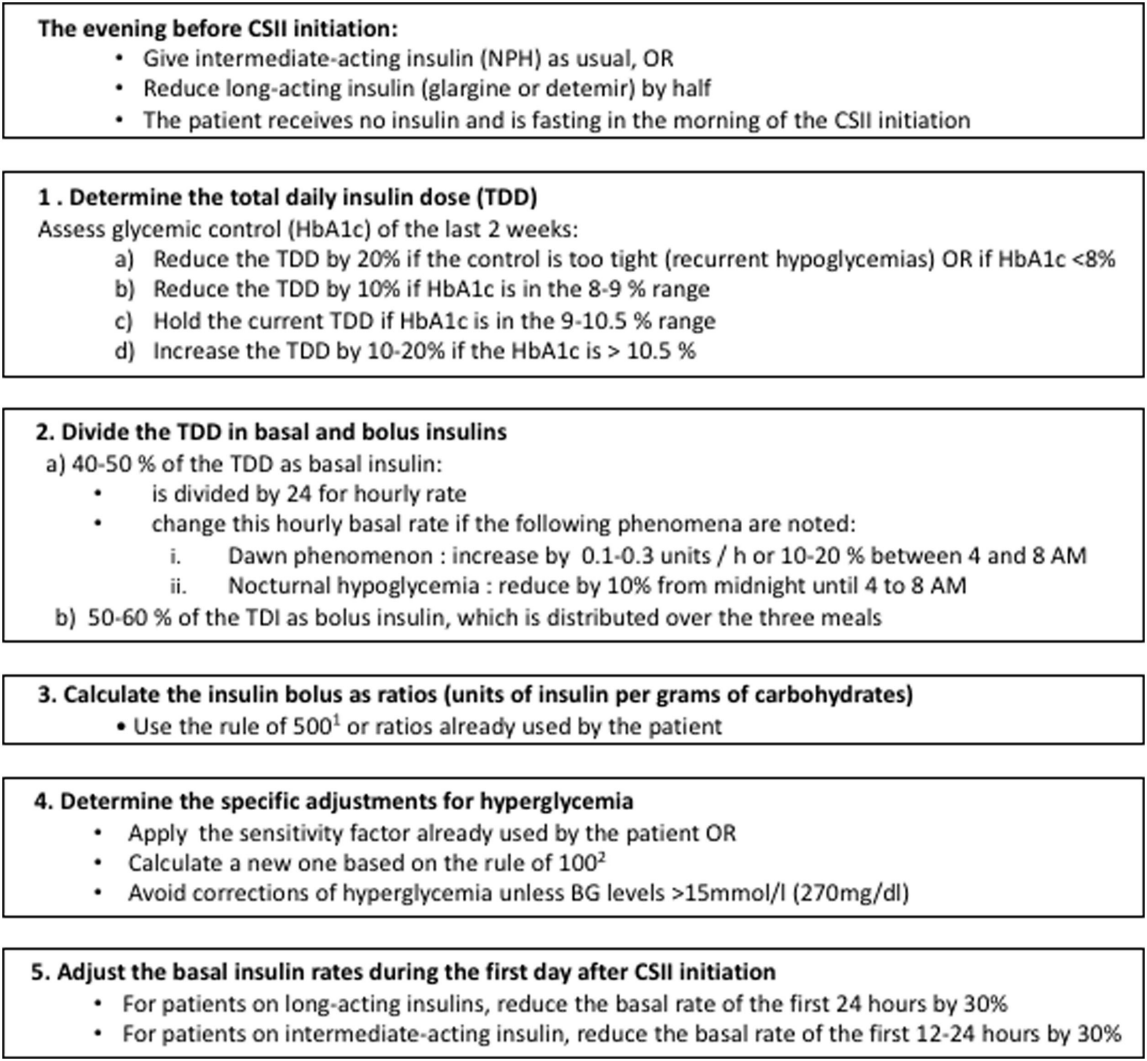
**CHU Sainte-Justine protocol for calculation of insulin dose at continuous subcutaneous insulin infusion (CSII) initiation**. ^1^Rule of 500 [used to calculate the carbohydrate factor (grams of carbohydrate per unit of insulin)]: 500 divided by the TDI (11). ^2^Rule of 100 (used to calculate the sensitivity factor): sensitivity factor equal to 100 divided by the total daily insulin (TDI) dose (11).

An increasing number of patients with type 1 diabetes mellitus (T1DM) under insulin injections will switch to CSII treatment during childhood (10). When transitioning to CSII, the medical team programs manually the initial parameters, according to the total daily insulin (TDI) dose and the current metabolic control of the patient. Next, the medical team adjusts the CSII parameters to reach an optimal glycemic control. Our local protocol for calculating the initial parameters of CSII therapy is shown in Figure 1.

To date, there is no consensus on the initial insulin doses recommended when starting CSII; this practice varies between centers and depends on the local experience (12). We identified several adult and pediatric protocols in the literature (12–21), with additional guidelines published by the American Diabetes Association,^1^ in textbooks (22, 23), by tertiary centers,^2^ and by pharmaceutical manufacturers.^3^ However, to our knowledge, none of these protocols has been validated for safety in children in a prospective study.

When comparing the above protocols, we observe large variability in the method of calculation. While most of them propose a reduction in the TDI dose while transitioning to CSII, the insulin dose of our protocol is relatively lower. Although, as mentioned above, glycemic control with long-term CSII use has been extensively studied in the literature (1–9), only one pediatric study has retrospectively assessed insulin needs at CSII initiation without reporting in detail rates of hypoglycemia and hyperglycemia (18).

In this context, we designed a prospective, single-center study. The main objective was to assess the difference in the frequency of hypoglycemia before and after initiation of CSII. We hypothesized that the frequency of hypoglycemia would be increased in the first 3 days after initiation of CSII compared to the 3 days prior to initiation. A secondary objective was to evaluate the overall glycemic control in the first days after CSII initiation, taking into consideration both hypoglycemia and hyperglycemia rates. For both outcomes, we aimed to explore the interaction of potential confounders [age, sex, body mass index (BMI), duration of T1DM, TDI, HbA1c, and type of insulin regimen before CSII].

## Materials and Methods

### Protocol

The eligibility of patients starting on CSII was defined according to the following inclusion criteria: (1) patients diagnosed with T1DM and followed at our center; (2) insulin needs of >0.5 U/kg/day at the time of enrollment, to avoid the honeymoon period (24, 25); (3) most recent value of HbA1c between 7 and 10% (5th and 85th percentiles of our population), to exclude patients prone to repeated hypo- or hyperglycemia; (4) patients living at a distance of less than 50 km from our center (to enable visits at home by the research nurse); and (5) informed written consent prior to the entry in the study.

Before starting on CSII, all patients and their parents attended a series of outpatient training courses. During one of these courses, a week prior to CSII initiation, they received written information explaining the study. The eligible patients and their parents were then contacted by a research nurse, and those willing to participate were enrolled in the study only after a written informed consent was obtained by the patients and the parents of all non-adult patients.

As part of this project, we required the use a continuous glucose monitoring (CGM) device [iPro^®^2 CGM System (Medtronic MiniMed, Northridge, CA, USA)] for 6 days (3 days prior and 3 days after insulin pump initiation). This type of censor is “blinded” to the patient [no possibility of instantaneous reading of the blood glucose (BG) values]. Thus, the patient cannot adjust the insulin doses according to the CGM measures using this device. The CGM sensors were installed at the participant’s home by a research nurse. Then, participants were asked to continue measuring their BG levels using their meter as usually recommended, or a minimum of four times a day prior to, and a minimum of eight times per day after the CSII initiation. No changes in diet and exercise habits were required prior to CSII start. Participants were asked to document the following information: injections of bolus insulin, meals, physical activity, and hypoglycemia (symptomatic or severe). As typically recommended at CSII initiation, we required to avoid snacks containing carbohydrates and moderate to intense exercise that could change the basal metabolism during the first 3 days on CSII. CGM data were considered valid if we obtained a total recording time of at least 24 h before and 24 h after pump initiation.

To be consistent, all changes in insulin doses were performed according to the following rules: (i) in cases of hypoglycemia, clinicians were instructed to wait for three consecutive episodes of hypoglycemia below 4 mmol/L (72 mg/dL) before decreasing the basal rate by 20% until the next morning at 8:00 a.m.; (ii) during episodes of hyperglycemia, we recommended to measure ketonemia if glucose values were >15 mmol/L (270 mg/dL) and to contact the diabetes clinic if abnormal; and (iii) to facilitate CSII adjustments, correction doses were recommended only if BG values were >15 mmol/L before meals, according to a prescribed scale.

### Variables Analyzed

The following data were obtained from patients’ medical records: sex, age, weight, BMI, duration of T1DM, TDI, HbA1c prior to CSII initiation, previous type of insulin regimen, comorbidities, medications, and number of hospitalizations for severe hypoglycemia or ketoacidosis. HbA1c was measured by high-pressure liquid chromatography (Somagen Diagnostics Inc., Edmonton, AB, Canada).

Continuous glucose monitoring values were compared with values obtained by the glucometer to ensure the reliability of the data. CGM glucose levels were analyzed according to the following definition criteria: hypoglycemia was defined as CGM reading of <4 mmol/L and hyperglycemia as CGM reading of >15 mmol/L. Prolonged episodes (of either hypoglycemia or hyperglycemia) were defined as episodes of abnormal values lasting >1 h, corresponding to ≥12 consecutive abnormal CGM glucose readings (the iPro^®^2 CGM automatically records an average glucose value every 5 min).

### Data Analysis

The rate of occurrence of events was defined as the number of abnormal CGM glucose readings (<4 or >15 mmol/L) divided by the total number of CGM readings available for the period (i.e., 3 days pre- and post-installation of the CSII); the result was then expressed as number of abnormal readings per person-reading. For each participant, we compared the above results before and after CSII initiation, and their ratio was expressed as relative rate (RR). The same type of analysis was followed to compare rates of prolonged episodes of hypoglycemia and hyperglycemia before and after CSII initiation. A detailed description of the statistical tests used in our comparisons appears in Section “Appendix.”

## Results

### Recruitment

**Figure 2:**
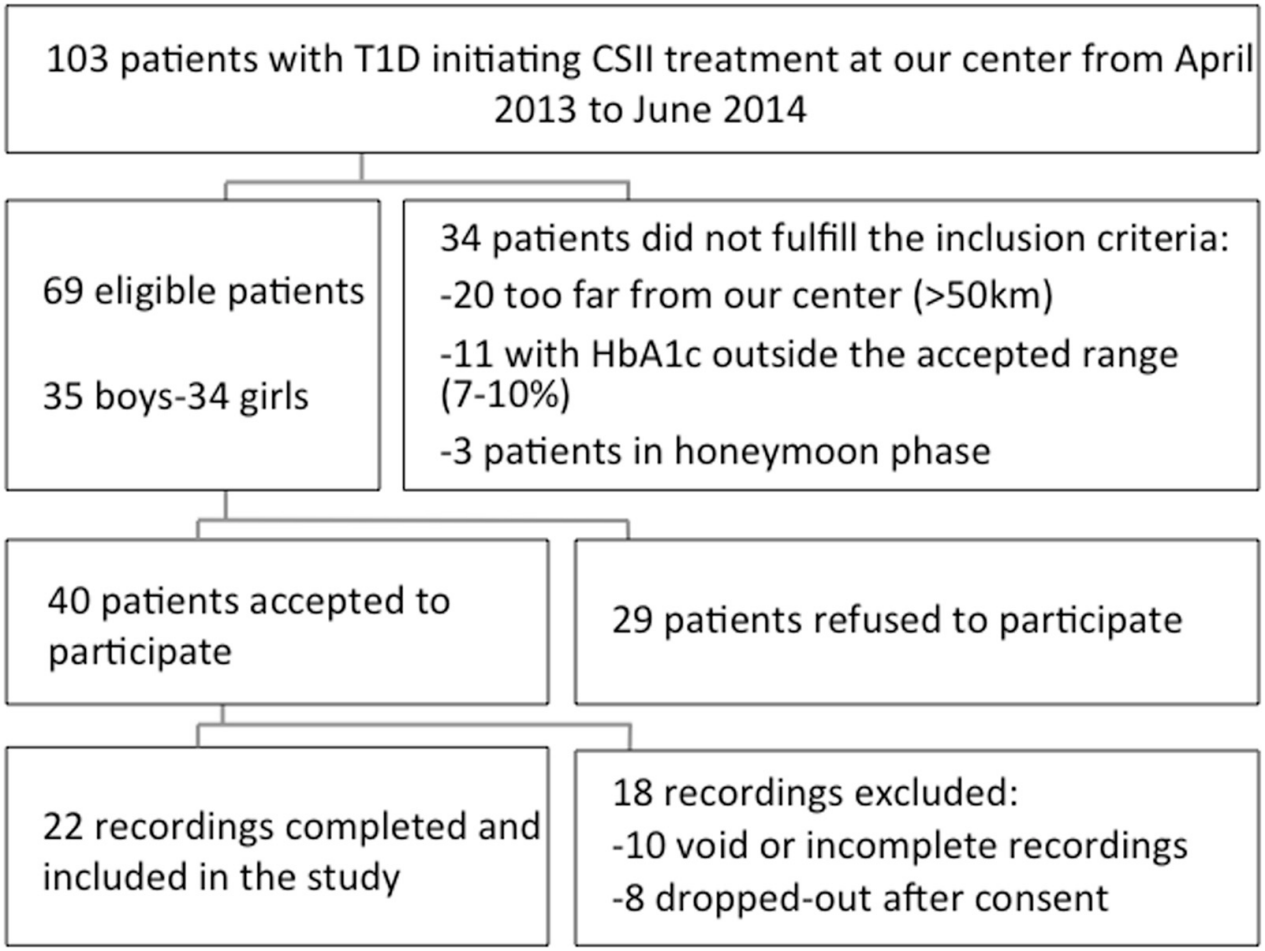
**Breakdown of study subjects through inclusion and exclusion criteria**.

Among 103 patients with T1DM starting on CSII at our clinic between April 2013 and June 2014, 69 subjects met the eligibility criteria (Figure 2). Among the 69 eligible patients, 40 subjects accepted to participate and 29 refused. The main reasons for these refusals were time investment and absence of direct benefit from participating. Among the 40 obtained CGM recordings, 22 were finally included in the analysis. Ten recordings were rejected due to limited number of readings: five were completely void due to various reasons (e.g., CGM device never captured BG values, low battery), and another five were incomplete due to a technical failure or accidental disconnection of the CGM device. Finally, eight subjects were considered as drop-outs: three participants with an initially installed CGM were excluded as their physician decided that they were not ready for the CSII, and the remaining five subjects removed the CGM due to discomfort generated by the device (skin reaction, pain, and itchiness).

### Characteristics of Participants and Non-Participants

Three groups were compared: participants who completed the study with appropriate CGM recordings (*n* = 22), participants who were excluded of the final analysis (insufficient CGM recordings or drop-outs, *n* = 18), and patients who refused to participate (*n* = 29). The baseline characteristics of the three groups did not differ significantly. None of the 22 participants had a history of celiac or Addison’s disease, or previous hospitalizations for episodes of severe hypoglycemia or ketoacidosis.

Baseline characteristics of participants previously on long-acting insulin (*n* = 12) and intermediate-acting insulin (*n* = 10) are shown in Table 1. Only duration of diabetes was different between the two groups, with the long-acting insulin group having a longer duration of diabetes (71.5 vs 16 months, *p* = 0.001). This difference was not surprising, given that the majority of patients with newly diagnosed T1DM in our center start with an intermediate-acting insulin and switch to long-acting insulin later during their follow-up.

**Table 1:** **Comparison of baseline characteristics of the long-acting insulin group vs intermediate-acting insulin group**.

Baseline characteristics	Long-acting insulin group (*n* = 12)	Intermediate-acting insulin group (*n* = 10)	*p*-Value
Male [*n* (%)]	6 (50%)	4 (40%)	0.29
Age (years)	13.36 ± 3.72	10.90 ± 4.61	0.19
BMI (kg/m^2^)	19.58 ± 3.24	18.64 ± 2.91	0.48
Duration of diabetes (months)	71.5 (13–122)	16.0 (7–30)	0.001*
TDI dose (U/kg/day)	0.80 (0.54–1.66)	0.65 (0.45–0.96)	0.1
HbA1c (%) (mmol/mol)	8.25 ± 1.05	7.83 ± 0.90	0.32
	66.7 ± 11.5	62.1 ± 9.90	

*P values below 0.05 are denoted by an asterisk.

*BMI, body mass index; TDI, total daily insulin*.

*Values are numbers and %, means ± SD, or median (range)*.

### Episodes of Prolonged Hypoglycemia and Hyperglycemia

The average number of CGM glucose readings per patient was 693 ± 220 before CSII and 837 ± 63 after the CSII initiation. When we compared the values before and after CSII initiation, we found no difference in the rate of incidence of abnormal readings, in both hypoglycemic and hyperglycemic ranges, and in the number of prolonged episodes of hypoglycemia and hyperglycemia per day of recording (Table 2).

**Table 2:** **Frequency of abnormal readings and of prolonged episodes before and after continuous subcutaneous insulin infusion (CSII) initiation**.

Variable		Before CSII initiation	After CSII initiation	*p*-Value
Abnormal readings per total person-readings (%)	Hypoglycemia	8.9 (0–18.6)	3.9 (0–31.9)	0.52
	Hyperglycemia	10.6 (0–37.8)	15.4 (0–48.2)	0.31
Number of prolonged episodes (>1 h) per day	Hypoglycemia	1.0 (0–3.8)	1.0 (0–3.6)	0.57
	Hyperglycemia	0.9 (0–5.2)	1.6 (0–5.8)	0.06

*Values are expressed as median (range)*.

Also, there was no increase of the RR of prolonged hypoglycemic episodes after CSII initiation (Table 3). None of the participants presented severe hypoglycemia. Only one participant, formerly under long-acting insulin (Glargine^®^), required an insulin dose adjustment during the first 24 h after pump initiation, because of persistent hypoglycemia, which did not recur after the above adjustment. However, there was a trend to an increase of prolonged hyperglycemic episodes [RR = 1.52 (95% CI 0.99–2.35), *p* = 0.058], with a median number of episodes per day of 0.9 before vs 1.6 after CSII initiation. None of the participants reported abnormally high blood ketones during the study duration. When we adjusted for potential confounders (sex, age, BMI, TDI, HbA1c, and type of insulin regimen before CSII), the increase of prolonged hyperglycemic episodes became statistically significant [RR = 1.61 (95% CI 1.05–2.50), *p* = 0.03] in the adjusted model.

**Table 3:** **RRs of abnormal readings and of prolonged episodes of hypoglycemia and hyperglycemia**.

Variable		RR^b^ unadjusted		RR^b^ adjusted for potential confounders^a^	
		RR^b^ (95% CI)	*p*-Value	RR^b^ (95% CI)	*p*-Value
Abnormal readings	Hypoglycemia	0.85 (0.52–1.39)	0.52	0.80 (0.49–1.32)	0.38
	Hyperglycemia	1.26 (0.81–1.97)	0.31	1.32 (0.87–2.01)	0.19
Prolonged episodes (>1 h)	Hypoglycemia	1.12 (0.75–1.69)	0.56	1.08 (0.71–1.65)	0.71
	Hyperglycemia	1.52 (0.99–2.35)	0.06	1.61 (1.05–2.50)	0.03

*^a^Adjusted for sex, age, BMI, TDI dose, HbA1c, and type of insulin regimen before CSII*.

*^b^RRs after CSII/before CSII. Ratios >1.0 indicate higher risk compared to before CSII*.

*RR, relative rate; BMI, body mass index; TDI, total daily insulin; CSII, continuous subcutaneous insulin infusion*.

## Discussion

The main objective of the present study was to evaluate the capacity of our CSII initiation protocol to prevent hypoglycemia at CSII initiation in children. To our knowledge, this is the first systematic prospective assessment of a protocol of insulin dose calculation at pump initiation. For this purpose, we used CGM to obtain BG readings before and after the start of CSII in the same patient. Therefore, each one of the 22 participants our study represents his/her own control. We considered this approach as a reliable method of comparing rates of hypoglycemia before and after CSII initiation.

Pediatric clinical studies implying additional burden (intervention such CGM device installation and time commitment) and few benefits have a notorious high refusal rate. Therefore, our present 42% refusal rate, while considered as a limitation of our study, is not surprising and is consistent with rates reported in previous pediatric studies (up to 51%), although the latter studies required up to 6 months of real-time-CGM (RT-CGM) utilization (26, 27). We observed a high rate of unsuccessful recordings obtained with the use of a CGM, with only 57% of successful recordings. Similar problems, related to both standard CGM and RT-CGM use, have been described in the pediatric and adult population (28–30). Finally, our 20% drop-out rate (8 out of 40 patients) is equal to the ones reported in 2 pediatric studies requiring long-term CGM use (28, 31).

Our results indicated that there was no evidence of increase of hypoglycemia after CSII initiation. In contrast, an increase of hyperglycemia after CSII initiation was observed, which was more significant in the group receiving long-acting insulin. This finding was probably the consequence of the recommended 50% reduction of the dose of long-acting insulin at bedtime the day before CSII installation, whereas a decrease of the intermediate-acting insulin dose was not recommended. Of note, we did not allow supplemental correction doses in the early days after pump installation for BG below 15 mmol/L; the aim was to better understand the BG values and to better adjust the boluses and the basal rates. Furthermore, patients with newly installed CSII were asked to avoid moderate to intense exercise, which may explain the higher incidence of hyperglycemia in the days following the CSII initiation. Despite the increase in the episodes of prolonged hyperglycemia, the absence of reported high blood ketone levels during these episodes was reassuring in regards to the safety of our approach.

Our study has several limitations. First, given the lack of previous comparable studies, a proper power calculation to estimate the required number of patients to enroll was not possible. In this regard, our project should be considered as a pilot study. With the collection of a large number of glucose values per individual, our study had enough power to detect differences in the rates of prolonged hyperglycemia, and this suggests that the absence of difference in prolonged hypoglycemia is real and not due to missed true differences. Second, the CGM devices can fail to detect hypoglycemia. Many studies in adults suggest that the accuracy of the CGM data in the hypoglycemic range may be less optimal in T1DM patients (32, 33). According to Rebrin et al. (34), current sensors are generally less accurate in the first 24 h due to local tissue inflammation following trauma at the time of insertion, leading eventually to an underestimation of hypoglycemia in the first day after CGM initiation. To mitigate this risk, we decided to install the CGM device already 3 days before the CSII initiation. Moreover, we did not observe less hypoglycemia on the first day of the 22 recordings available for analysis. Another limitation of our study is the heterogeneity in the age of the participants. Indeed, differences in diurnal patterns in insulin needs have been reported in different age groups (13). While the relatively small sample size of this study could not allow for stratification analysis, our findings suggest that our protocol is safe when applied in patients of different age groups. Finally, it is important to emphasize that our results, based on an observation period of 3 days after CSII initiation, are obviously not predictive of subsequent glycemic control and can only be used as a tool for initiation of CSII therapy.

## Conclusion

Our pilot study is the first to assess, through CGM, and in a prospective way, the impact of a CSII installation protocol on glycemic values in the first days after CSII initiation. Despite the relatively increased frequency of hyperglycemia, our protocol does not lead to clinically relevant hypoglycemia and provides a safe model of calculating insulin doses for CSII in children. Further studies in larger numbers of patients, allowing for the evaluation of other protocols or the adaptation of the current one are needed, while new features in CSII devices, such as low glucose suspend, may further improve the safety at CSII initiation.

## Ethics Statement

This project was approved by “the Research Ethics Committee of the CHU Sainte-Justine,” and all research procedures were conducted in accordance with the recommendations of this Committee. All subjects gave written informed consent in accordance with the Declaration of Helsinki. This study was registered on www.clinicaltrials.org (NCT01840358). The protocol was approved by the “Commite d’Ethique du CHU Sainte-Justine.”

## Author Contributions

DM contributed to the study concept and design, collected data, supervised the study, participated in data analysis and interpretation, and drafted, reviewed, and edited the manuscript. PO contributed to the study concept and design, collected data, supervised the study, participated in data analysis and interpretation, and reviewed and edited the manuscript. JD contributed to the study concept and design, participated in data analysis and interpretation, and reviewed and edited the manuscript. LG contributed to the study concept and design as well as reviewed and edited the manuscript.

## Conflict of Interest Statement

The authors declare that the research was conducted in the absence of any commercial or financial relationships that could be construed as a potential conflict of interest.

## Footnotes

^1^http://www.diabetes.org/living-with-diabetes/treatment-and-care/medication/insulin/getting-started.html

^2^http://www.nhslanarkshire.org.uk/Services/Diabetes/Diabetes%20Clinical%20Guidelines/Documents/Insulin%20Pump%20Therapy%20May%2014.pdf

^3^http://www.professional.medtronicdiabetes.com/resources-download-library

## Appendix

### Statistical Methods

The rate of occurrence of hyperglycemic and hypoglycemic events was defined as the number of abnormal continuous glucose monitoring (CGM) glucose readings divided by the total number of CGM readings available for the period; the result was then expressed as number of abnormal readings per person-reading. The comparison of these results was expressed as a relative rate (RR), where the numerator is the number of abnormal readings per total number of person-readings after continuous subcutaneous insulin infusion (CSII) initiation, and the denominator is the number of abnormal readings per total number of person-readings before CSII initiation. The prolonged episodes of hypoglycemia and hyperglycemia (>1 h) are expressed as number of episodes per day of recording. The RR for the prolonged hypoglycemic and hyperglycemic episodes represents the ratio of their incidence post-CSII to the incidence pre-CSII, respectively. As it was a paired test (the participant was his/her own control), all these variables were analyzed by a Poisson GEE with a “cluster” effect for the subject. Parameters that represented potential confounding variables (sex, age, body mass index, total daily insulin, HbA1c, and type of insulin regimen before CSII) were then added in the Poisson GEE model and, when the model indicated a possible interaction (defined as a level of significance corresponding to a *p* < 0.1), a subgroup analysis was performed. Finally, for other analyses, we used Student’s *t*-test for the normally distributed values, chi-squared to compare proportions, and ANOVA and Kruskal–Wallis for multiple comparisons. The analyses were performed using the SPSS 21, and *p*-values <0.05 were considered significant.
